# Identification of a potent small molecule capable of regulating polyploidization, megakaryocyte maturation, and platelet production

**DOI:** 10.1186/s13045-016-0358-y

**Published:** 2016-12-08

**Authors:** Nick Huang, Mabel Lou, Hua Liu, Cecilia Avila, Yupo Ma

**Affiliations:** 1 Department of Pathology, State University of New York, Stony Brook Medicine, Stony Brook, NY 11794 USA; 2 Department of Obstetrics & Gynecology, State University of New York, Stony Brook Medicine, Stony Brook, NY USA

## Abstract

**Background:**

Megakaryocytic cell maturation involves polyploidization, and megakaryocyte (MK) ploidy correlates with their maturation and platelet production. Retardation of MK maturation is closely associated with poor MK engraftment after cord blood transplantation and neonatal thrombocytopenia. Despite the high prevalence of thrombocytopenia in a range of setting that affect infants to adults, there are still very limited modalities of treatment.

**Methods:**

Human CD34^+^ cells were isolated from cord blood or bone marrow samples acquired from consenting patients. Cells were cultured and induced using 616452 and compared to current drugs on the market such as rominplostim or TPO. Ploidy analysis was completed using propidium iodide staining and flow cytometry analysis. Animal studies consisted of transplanting human CD34^+^ cells into NOD.Cg-Prkdc^scid^Il2rg^tm1Wjl^/SzJ mice followed by daily injections of 15 mg/kg of 616452.

**Results:**

Within one week of culture, the chemical was able to induce polyploidization, the process required for megakaryocyte maturation with the accumulation of DNA content, to 64 N or greater to achieve a relative adult size. We observed fold increases as high as 200-fold in cells of 16 N or greater compared to un-induced cells with a dose-dependent manner. In addition, MK differentiated in the presence of 616452 demonstrated a more robust capacity of MK differentiation than that of MKs cultured with rominplostim used for adult idiopathic thrombocytopenic purpura (ITP) patients. In mice transplanted with human cord blood, 616452 strikingly enhanced MK reconstitution in the marrow and human peripheral platelet production. The molecular therapeutic actions for this chemical may be through TPO-independent pathways.

**Conclusion:**

Our studies may have an important impact on our fundamental understanding of fetal MK biology, the clinical management of thrombocytopenic neonates and leukemic differentiation therapy.

**Electronic supplementary material:**

The online version of this article (doi:10.1186/s13045-016-0358-y) contains supplementary material, which is available to authorized users.

## Background

Megakaryocytes are one of few cell types that undergo endomitosis, a form of cell cycle that skips the late stages of mitosis to become polyploid [1–4]. Human megakaryocytes commonly reach ploidy states of 16 N and can achieve states as high as 128 N. The mechanism of polyploidization is still not well understood, however, polyploidy is required for functional human megakaryocyte maturation. Once active, the megakaryocytes are responsible for the production of platelets that have well-characterized roles in hemostasis, thrombosis, vascular integrity, development of the lymphatic system, and the innate immune response [5–8].

Thrombocytopenia affects approximately 20–35% of infants admitted to the neonatal intensive care unit [9–11]. Approximately 9% of those infants are severe and experience clinically significant bleeding (usually intracranial). Platelet transfusions are one of the only therapeutic options for thrombocytopenic neonates. Recent studies have shown that megakaryocytes of neonates are smaller and have lower ploidy than those of adults [12, 13]. Small megakaryocytes usually produce fewer platelets than large megakaryocytes and typically achieve adult size at approximately 1 year of age. Therefore, an inability to increase megakaryocyte size and ploidy in response to increased platelet consumption might underlie the predisposition of sick neonates to thrombocytopenia.

In adults, clinically significant thrombocytopenia is often multifactorial often involving cytotoxic or suppressive effects of chemotherapeutic agents and malignant cells, respectively. Thrombopoietin (TPO) is synthesized in the liver and is the primary regulator of megakaryocyte development and maturation [14, 15]. Recombinant human TPO (rhTPO) has been shown to attenuate carboplatin-induced thrombocytopenia, reducing the need for platelet transfusions [16]. However, the clinical development of rhTPO has since been halted due to the natural development of anti-TPO antibodies in patients. Alternative routes to target TPO receptors such as eltrombopag, a non-peptide, small molecule, that have been shown to stimulate megakaryopoiesis of CD34^+^ cells in patients with multiple myeloma are in the pipelines [17, 18].

Human umbilical cord blood (hUCB) is an important stem cell source for patients who lack other suitable donors. However, slower platelet engraftment is a major drawback of hUBC transplantation. Platelet engraftment takes an average of approximately 50 days for hUBC recipients, versus 20 days for mobilized peripheral blood cells derived from adult donors [12]. Identification of a megakaryocyte maturation inducer or co-transfusion of large numbers of ex vivo generated human megakaryocyte-committed cells with high maturation potential, could provide an alternative method to shorten period of thrombocytopenia [19]. TPO and its derivatives have been used in the treatment of thrombocytopenia in adult but not neonatal patients. However, studies in models using the non-human primate or canine demonstrated that standard post-transplant admiration of TPO could not accelerate platelet reconstitution following autologous bone marrow transplantation (AuBMT ) or allogenic bone marrow transplantation (alloBMT), respectively, in myeloablated hosts [20–23]. TPO stimulates the megakaryocyte formation in vivo, but it does not shorten its maturation time [22].

Although the cellular and molecular mechanisms underlying the differences of neonatal and adult MKs remain unclear, studies in congenital disorders have begun to elucidate these mechanisms. A transient myeloproliferative disorder with immature MK features (impaired maturation of MKs) is seen exclusively in fetuses and neonates with Down syndrome and GATA1 mutations indicating that thrombopoietin (TPO)-independent pathways may play a critical role in neonatal/fetal MK maturation [22, 24, 25].

In this manuscript, we introduce and characterize a novel chemical that has not yet been implicated in megakaryopoiesis. We found that this chemical molecule selectively increased polyploidization and shortened maturation of cord blood MKs. The size and ploidy of cord blood/adult mobilized peripheral blood megakaryocytes were also dramatically increased in response to this chemical small molecule stimulation with a dose-dependent fashion. The chemical also induced human peripheral platelet production in mice transplanted with human cord blood. The molecular action of this chemical to stimulate the shortened MK maturation may be through TPO-independent pathways.

## Methods

### Isolation of CD34^+^ cells from mobilized peripheral blood, bone marrow, or umbilical cord blood

Cells were isolated with MACS CD34^+^ microbead kit following manufacturer protocols. In the isolation of cord blood, cells underwent a lysis phase for 15 min at room temperature using BD PharmLyse prior to undergoing the MACS CD34^+^ isolation protocol. Percentage of viable CD34^+^ obtained ranged from 90–98% in purity.

### HSC culture

Standard culture of CB cells used StemSpan SFEM (StemCell Technologies) containing 10% FBS (Gibco), 100 units/ml Pen Strep, 100 ng/ml SCF, TPO, and Flt-3 ligand (Peprotech). For expansion of cells targeted for cell sorting, we used the same media containing a different cytokine cocktail of 100 ng/ml SCF, TPO, and 10 ng/ml IL-3.

### Chemicals

616452 and 616454 were purchased from Calbiochem. SB431542 was purchased from Tocris. All chemicals were reconstituted according to manufacturers’ instructions. For animal studies, the 616452 was purchased in bulk from BioVision.

### Ploidy Analysis

Cells were centrifuged for 5 min at 200*g* at 4 °C. Pellet was resuspended in 70% ethanol for overnight at 4 °C. After which, the cells were washed with PBS and centrifuged for 5 min at 200*g* at 4 °C. The pellets were resuspended in PI staining buffer containing PBS with 1% Triton X-100, 100 ul of 2 mg/ml PI, and 0.25 ug/ml of deoxyribonuclease (DNAse)-free ribonuclease (RNAse). The following suspension was incubated at room temperature for 30 min prior to analysis.

### Flow cytometry analysis

Cells were centrifuged for 5 min at 200*g* at 4 °C. Cells were resuspended with CD34, 38, and/or 41 conjugated antibody for 30 min on ice. Cells were then washed with 2 ml of PBS and centrifuged for 5 min at 200*g* at 4 °C. The final pellet was resuspended using 300 ul of 2% formalin. Analysis was run on the FACSCalibur.

### Flow–cell sorting

Cells were centrifuged for 5 min at 200*g* at 4 °C and resuspended with 25 ul of CD41-FITC antibody for 30 min on ice. Cells are then washed gently with PBS and centrifuged for 5 min at 200*g* at 4 °C. Cells are then suspended at a concentration of 10^6^ cells/ml of PBS containing 2% FBS. Cells are sorted via FACSAria.

### Animal study: platelet recovery upon injection of chemical

Twelve 10-week-old NOD.Cg-Prkdc^scid^Il2rg^tm1Wjl^/SzJ (Jackson Laboratories) male mice were given sublethal irradiation of 2.25 Gy. The following day, mice were given 50000 CD34^+^ cells and 300000 progenitor cells (CD34^+^ cells that were cultured for 4 days) via IV injection. Mice were given daily 616452 injections at 15 mg/kg. Mice were bled via submandibular bleeding every 3–4 days, and the collected blood was analyzed using the HemaVet 950 for platelet and cellular composition. The mice were sacrificed on day 12, and their bone marrow was analyzed for CD34 and CD41.

### Gene expression profile

Cells were induced using 616452 for 2 and 4 days with the control cells induced with dimethyl sulfoxide (DMSO). Cells were pelleted and flash frozen in liquid nitrogen and analyzed using PAHS-054Z Hematopoiesis array via Qiagen’s service center.

### RNA isolation and quantitative real-time polymerase chain reaction

Total RNA was isolated from human umbilical cord blood cells treated with or without 616452 using the AllPrep DNA/RNA Mini Kit (Qiagen Group). According to the manufacturer’s protocol, 1 ug total RNA from each sample was subjected to complementary DNA (cDNA) synthesis using QuantiTect® Reverse Transcription Kit (Qiagen Group). PCR was carried out using Power SYBRR Green PCR Master Mix (Applied Biosystems) and performed on a 7500-real-time PCR system (Applied Biosystems). The data were analyzed using the delta-delta Ct method.

## Results

### Identification of small molecules that induce polyploidization

Previously, we have shown that the stem cell gene SALL4 is a robust stimulator of expansion of hematopoietic stem/progenitor cells [26–28] and plays a critical role in hematopoietic differentiation. We screened small molecules targeting SALL4 expression using a SALL4 luciferase reporter. One TGF-β pathway inhibitor, 616452, was identified as a strong inducer of SALL4 expression. When cultured with isolated CD34^+^ cells, we noticed the appearance of large megakaryocyte-like cells.

### Induction of megakaryocyte differentiation and maturation in bone marrow (BM) CD34+

**Fig. 1:**
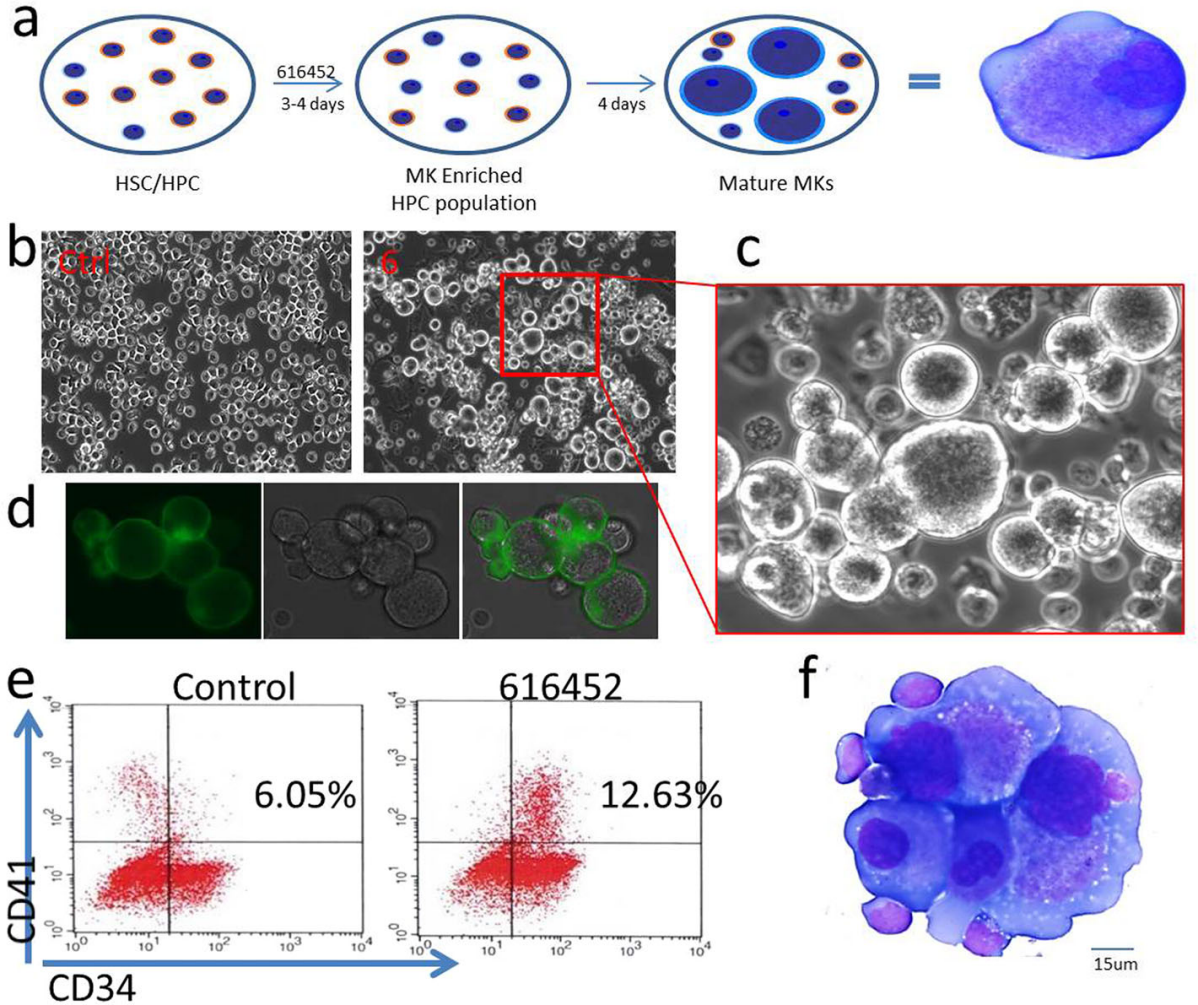
616452 induces megakaryopoiesis in human BM CD34^+^ cells. A representative plot of the effectiveness of the chemical 616452, and how the induction of megakaryopoiesis was induced (**a**). BM CD34^+^ cells were induced with 616452 (10 uM) for 8 days and compared to control (**b**, **c**). The induced cells were stained by live cell staining for CD41 (**d**) and analyzed by flow cytometry on day 5 (**e**). Cells were also collected for Giemsa-Wright staining after 10 days of culture (**f**), to illustrate the multi-nucleated and lobular nature of the nuclei

Initially, 616452 was identified as a MK inducer in BM CD34^+^ cells when a significant number of large megakaryocytes appeared within 4 days of culture. The control, induced with the compound solvent, only appeared to have one or two cells. By 10 days of culture, the large cells had taken over the dish (Fig. 1a–c). Live cell staining and immunofluorescence revealed all the large cells were positive for CD41, a unique marker of megakaryocytes [29] (Fig. 1d). Analysis by flow cytometry showed an increase of two-fold in the CD41^+^ CD34^+^ population within 4 days of culture (Fig. 1e). Giemsa-Wright staining revealed these cells are as large as 90 um with the average around 50 um (Fig. 1f). Although a dozen other TGF-β inhibitors have been described, 616452 was the only one we tested that strongly induced SALL4 stem cell gene expression and stimulated MK progenitor cell expansion and differentiation.

### Potent agonist of cord blood (CB) megakaryocyte differentiation and maturation from umbilical cord blood

Accumulated evidence demonstrates that neonatal MKs are significantly smaller, of lower ploidy, and produce fewer platelets than MKs from adults. Based on these characteristics, MKs from fetuses and neonates have been considered to be immature compared with adult MKs. We then tested the chemical effects on neonatal MK maturation. Cells isolated from human umbilical CB were cultured in hematopoietic stem cell (HSC) media induced with 616452 and compared DMSO-treated control cells. Larger cells characteristic of developing megakaryocytes appeared after 4 days of culture (Additional file 1: Figure S1a). The quantity and size of cells increased over time. By 8 days, the culture dishes were predominately composed of megakaryocytes with the formation of megakaryocytic clusters beginning. A Giemsa-Wright stain of these cells revealed a lobular multi-nucleated structure and a granulocytic cytoplasm characteristic of megakaryocytes (Additional file 1: Figure S1c). Flow analysis revealed an increase of 44.8% of the CD41^+^ population as well as an increase in the CD34^+^ CD41^+^ population (Additional file 1: Figure S1b) similar to that of bone marrow.

**Fig. 2:**
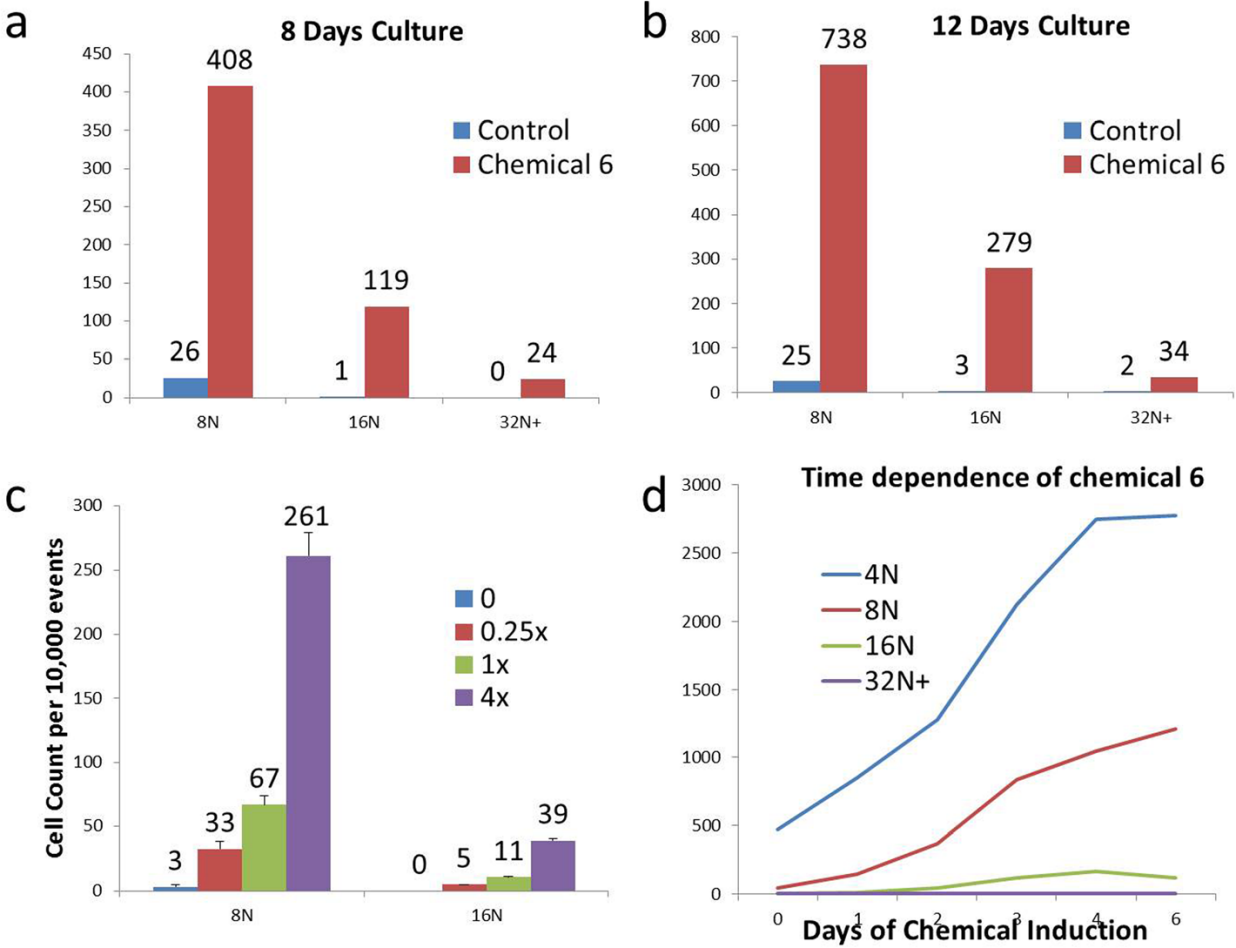
Dose and time dependence of 616452. Ploidy analysis of the MK’s derived from CB CD34^+^ cells after 8 and 12 days of culture (**a**, **b**). Cells were induced with 616452 (10 uM), and analysis were done on the respective days using PI staining and flow cytometry (*n* > 5). Dose-dependent analysis (*n* = 3) was done on CB CD34^+^ cells (**c**) with 1× concentration as 10 uM. Time-dependence studies (*n* = 3) used CB CD41^+^ cells that were isolated by flow cytometry (**d**). Cells were cultured for 6 days and chemicals were removed at certain time points and replaced with media containing only TPO. The control cells were grown entirely with TPO

One of the unique characteristics of megakaryocytes is their ploidy development, which can be easily measured using propidium iodide (PI), a fluorescent intercalating agent that binds nucleic acids. Cells induced with 616452 for 8 days revealed a drastically increased number of cells with greater ploidy. In humans, megakaryocytes normally account for approximately 0.05 to 0.1% of all nucleated bone marrow cells. Out of 10000 events, the control cells only elicited only 1 cell with a ploidy of 16 N and nothing greater (Fig. 2a). The chemically induced cells produced over 100 cells of 16 N ploidy and registered ploidy numbers as high as 64 N. 616452 consistently induced greater number of cells in each ploidy category 4 N and above. These studies were repeated by multiple independent experiments (*n* = 5).

### 616452 works independently of TPO

TPO has been highly associated with megakaryopoiesis, and one concern was whether this inhibitor is working in conjunction or is reliant on the presence of TPO. In the absence of TPO, there was limited cell growth, but MK development was still present with ploidy increase of 5–8-fold over the control in cells of 8 N or greater ploidy (Additional file 2: Figure S2g). In the presence of TPO, cells of 8 N ploidy increased dramatically more with a 55-fold increase over control. This likely suggests that 616452 is capable of functioning in a TPO-independent manner. However, it also has large synergistic interactions with TPO in accelerating the maturation of MKs.

### Time and dose dependence

Chemical induction of CB CD34^+^ revealed that the longer the cells were induced, the greater the ploidy development. Between 8 and 12 days of induction, the number of 8 N, 16 N, and 32 N cells nearly doubled (Fig. 2a, b), while the control remains almost unchanged. At 10 uM, chemical induces ploidy in CB CD34^+^ cells compared with control cells treated with TPO. Multiple replicates with CB from different individuals (*n* > 3) indicated that there is little or no 16 N ploidy in control cells. Cells treated with varying concentrations of chemical indicated dose dependence: 2.5 uM increased the numbers of 8 N ploidy cells by 10-fold compared to control, whereas 40 uM increased ploidy by 87-fold over the same population (Fig. 2c). There is also a time-dependent response with an increase in ploidy for up to 6 days in culture (Fig. 2d).

### MK differentiated in the presence of 616452 demonstrated a much robust capacity of MK differentiation than that of MKs cultured with Rominplostin (Amgen)

**Fig. 3:**
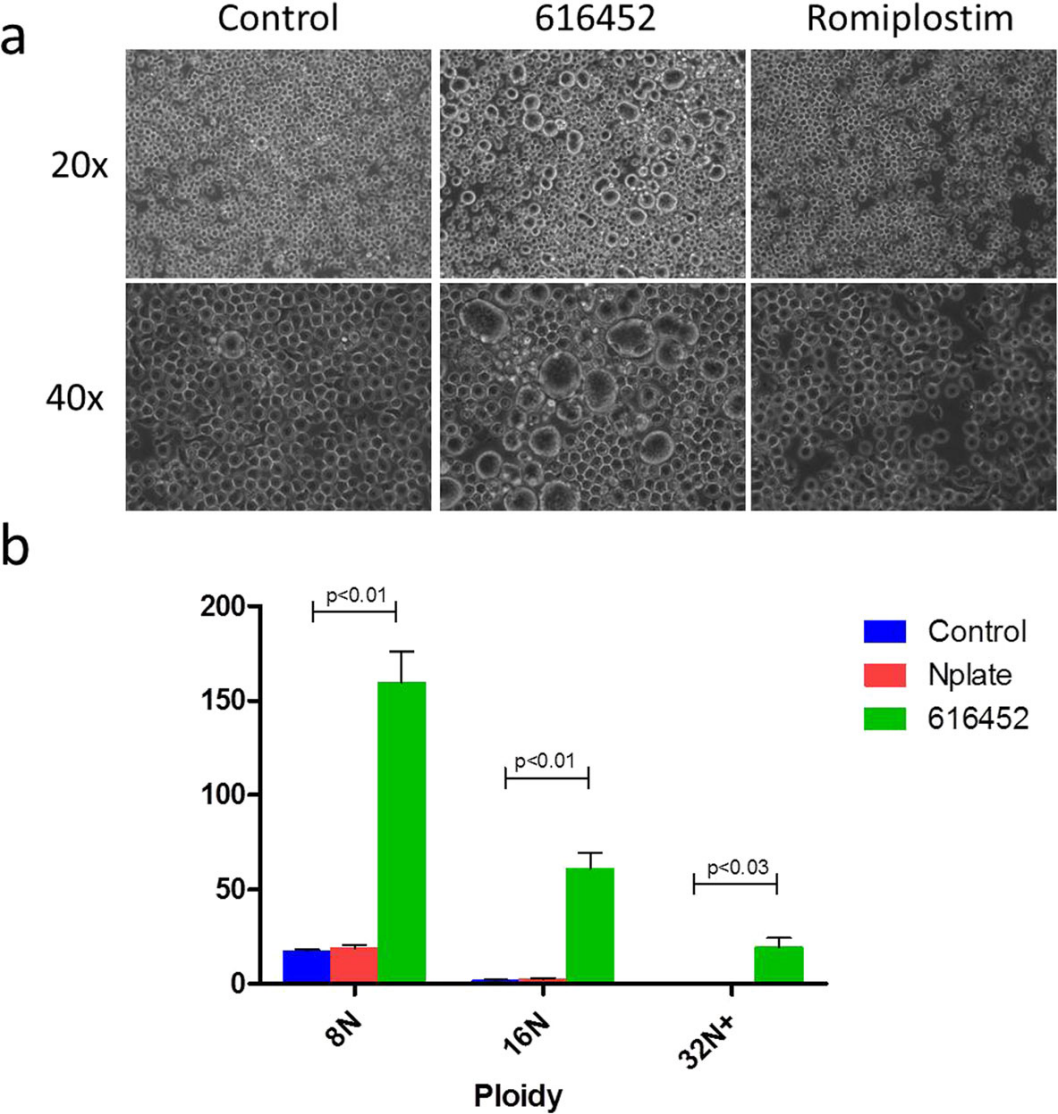
616452 on selectively isolated CD41^+^ cells CB CD34^+^ cells were cultured with TPO, SCF, IL-3 for 7 days prior for CD41 selection by flow cytometry (**a**). Control (DMSO induced) and chemical induced cells were grown(n=2) in StemSpan containing only TPO for 8 days prior to cell cycle analysis (**b**). In control cells we observed only 6% of the cells increased their ploidy numbers with no cells reaching a ploidy of 16N or greater, while 54% of the chemically induced cells had a nuclei of 4N or greater with a 4.5 fold increase in the number of 4N, 30 fold increase in 8N and the appearance of cells with even greater ploidy reaching as high as 64N within 8 days of culture (**c**, **d**). CB CD41^+^ cells were isolated using flow cytometry and cultured in the absence (**e**) or presence (**f**) of 616452(10uM). Cells appeared mostly as single cells on prior to day 6 (**e**, **f**) but began to form clusters thereon, while the control had little to none (**g**). By 8 days of induction, the clusters were large and covered the entire plate (**h**, **i**, **j**). A CD41 staining of these clusters illustrates the composition of these clusters as mainly large MK-like cells(**k**, **l**)

We observed a significant difference between the cells induced with 616452 compared to a current drug, romiplostim used for adult ITP patients. CB CD34^+^ co-cultured with 616452 showed numerous large MK cells within 8 days of culture as compared to that of rominplostim, which had little to none (Fig. 3a). The cells induced with romiplostim showed no significant difference from the control while cells induced with 616452 had an 8- and 30-fold increase in cells of 8 N and 16 N ploidy, respectively (Fig. 3b). The greater ploidy cells (32 N) could be seen both visually and phenotypically in the induced 616452 cultures which appeared drastically different than that of the control and romiplostim.

#### The extent of CD41^+^ megakaryocytic population in response to the induction of 616452

**Fig. 4:**
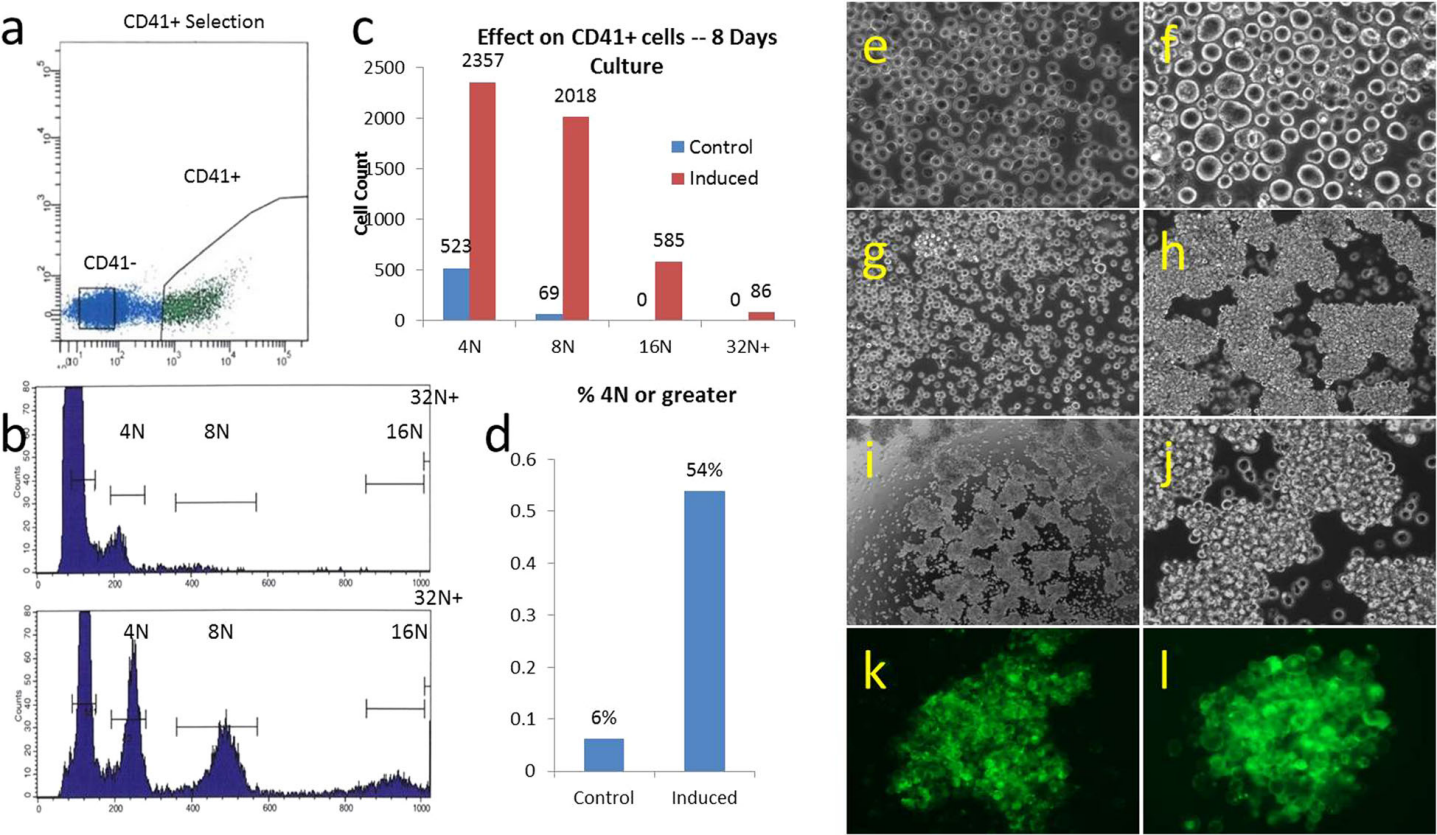
616452 on selectively isolated CD41^+^ cells. CB CD34^+^ cells were cultured with TPO, SCF, and IL-3 for 7 days prior for CD41 selection by flow cytometry (**a**). Control- (DMSO induced) and chemical-induced cells were grown (*n* = 2) in StemSpan containing only TPO for 8 days prior to cell cycle analysis (**b**). In control cells, we observed only 6% of the cells increased their ploidy numbers with no cell reaching a ploidy of 16 N or greater, while 54% of the chemically induced cells had a nuclei of 4 N or greater with a 4.5-fold increase in the number of 4 N, 30-fold increase in 8 N, and the appearance of cells with even greater ploidy reaching as high as 64 N within 8 days of culture (**c**, **d**). CB CD41^+^ cells were isolated using flow cytometry and cultured in the absence (**e**) or presence (**f**) of 616452(10 uM). Cells appeared mostly as single cell on prior to day 6 (**e**, **f**) but began to form clusters thereon, while the control had little to none (**g**). By 8 days of induction, the clusters were large (**h**) and covered the entire plate (**i**, **j**). A CD41 staining of these clusters illustrates the composition of these clusters as mainly large MK-like cells (**k**, **l**)

CB CD34^+^ cells were initially cultured for 8 days in a media specific to megakaryocyte expansion [30]. The cells were labeled with a conjugated CD41 antibody and sorted by FACS (Fig. 4a). The resulting CD41^+^ cells were induced with the small chemical, and cells began to increase their size within 2 days. By day 6, approximately 80% of the cells appeared to be large in size by eye while the control had little to none (Fig. 4e, f). Aggregation of the cells began around day 4 with clusters of about 5–15 cells, 25–50 cells by day 6 and by day 8 the majority of cells were in clusters of hundreds of cells. The control meanwhile had little clusters, but none that compared in size and numbers as the induced cells (Fig. 4g–j). A live cell stain revealed that 95% of the cells that were present after 8 days of induction with the small chemical were positive. The live cell stain also gave some insight into the structure of the large cell clusters as mainly comprised of large MKs (Fig. 4k, l). Out of 10000 events, a ploidy analysis of the cells after 8 days of induction revealed 2018 cells of 8 N, 585 cells of 16 N, and 86 cells of 32 N or greater ploidy (Fig. 4b, c). Approximately 54% of the cells had a ploidy number of 4 N or greater while the control cells induced with only TPO had only 6% of the cells with a ploidy of 4 N or greater (Fig. 4d). There were 69 cells with 8 N ploidy and nothing greater in the control cells. Meanwhile, in our small chemical induced cells, CD41-cells were also cultured under the same conditions with the control being induced using TPO and DMSO. There was little to no ploidy development in the control.

### Synergistic effect of combined TGF-β inhibition

**Fig. 5:**
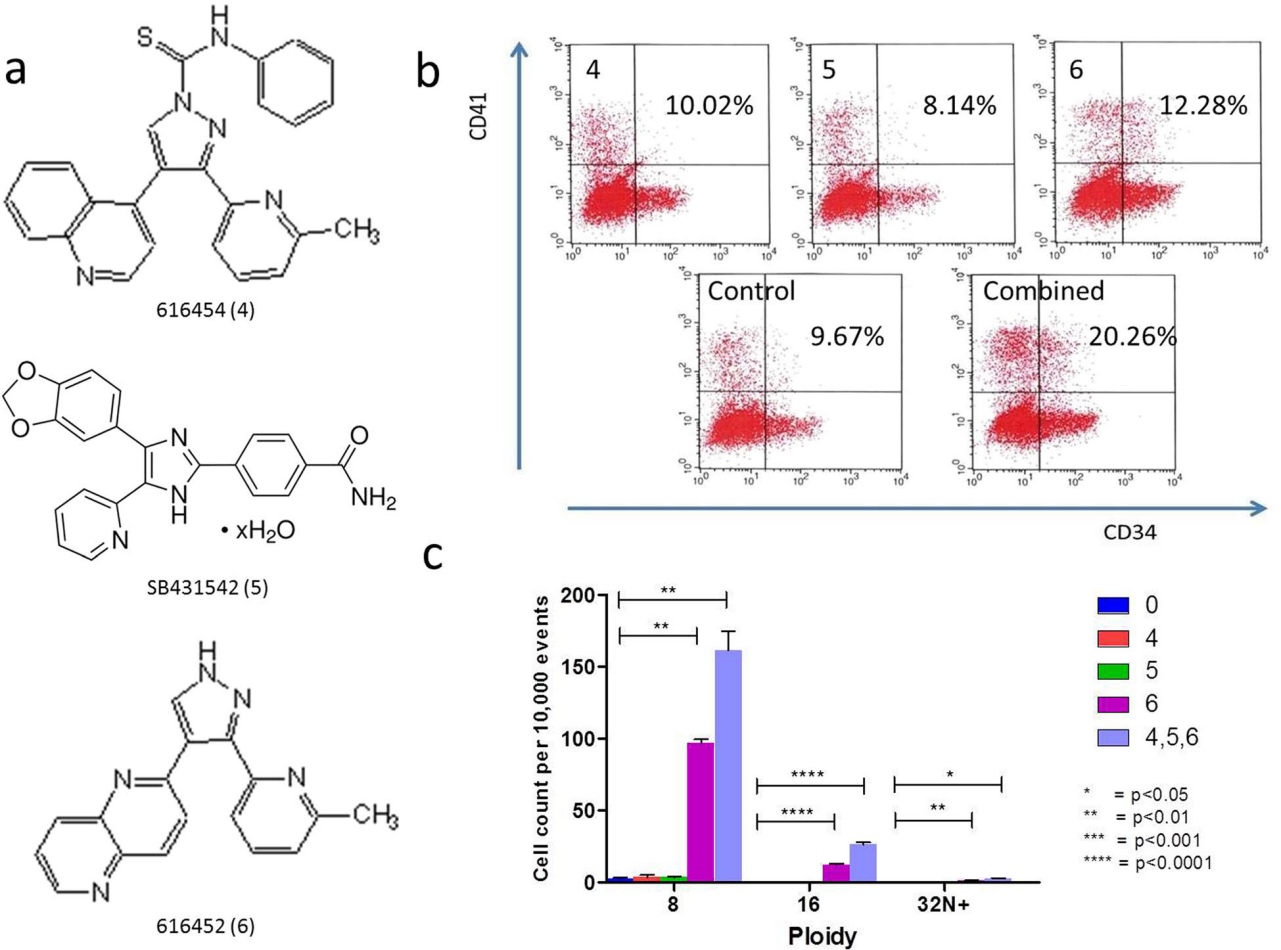
Comparison of 616452 against other TGF-β inhibitors. Various other TGF-β (**a**) were tested against 616452. Flow cytometry of CB CD34^+^ after 4 day induction (**b**) revealed inhibitors 616454 and SB431542 had little to no effect and may even negatively impact the differentiation into CD41^+^ cells while 616452 enriches the development by ~25%. The three TGF-b inhibitors induced at the following concentrations: 616454 (4) – 10uM, SB431542(5) - 10uM, 616452(6) – 10uM. When all three inhibitors combined, there is nearly a 100% increase in the enrichment. A ploidy analysis of the cells was run after 4 of induction the chemicals (**c**) to reveal an improved rate of maturation in terms of ploidy development on both days

Two additional TGF-β pathway inhibitors, 616454, and SB431542 (Fig. 5a), were tested for their ability to induce the expression of CD41 and/or the maturation of megakaryocytes via ploidy analysis. In both these cases, there were no observed increases in CD41 expression within 4 days, and no visible differences in cell size compared to control within 10 days of culture. A ploidy analysis revealed no significant deviation from the control with these two chemicals (Fig. 5b). However, when all these inhibitors were combined with 616452, we noticed a synergistic effect of chemicals on the expression of CD41 to a nearly 2-fold increase over the control (Fig. 5b, c) increasing the CD41 expression from 10.02 to 20.26%. Ploidy was also significantly affected with a 50 and 93% increase in cells of 8 and 16 N ploidy, respectively.

### Gene expression profile

**Fig. 6:**
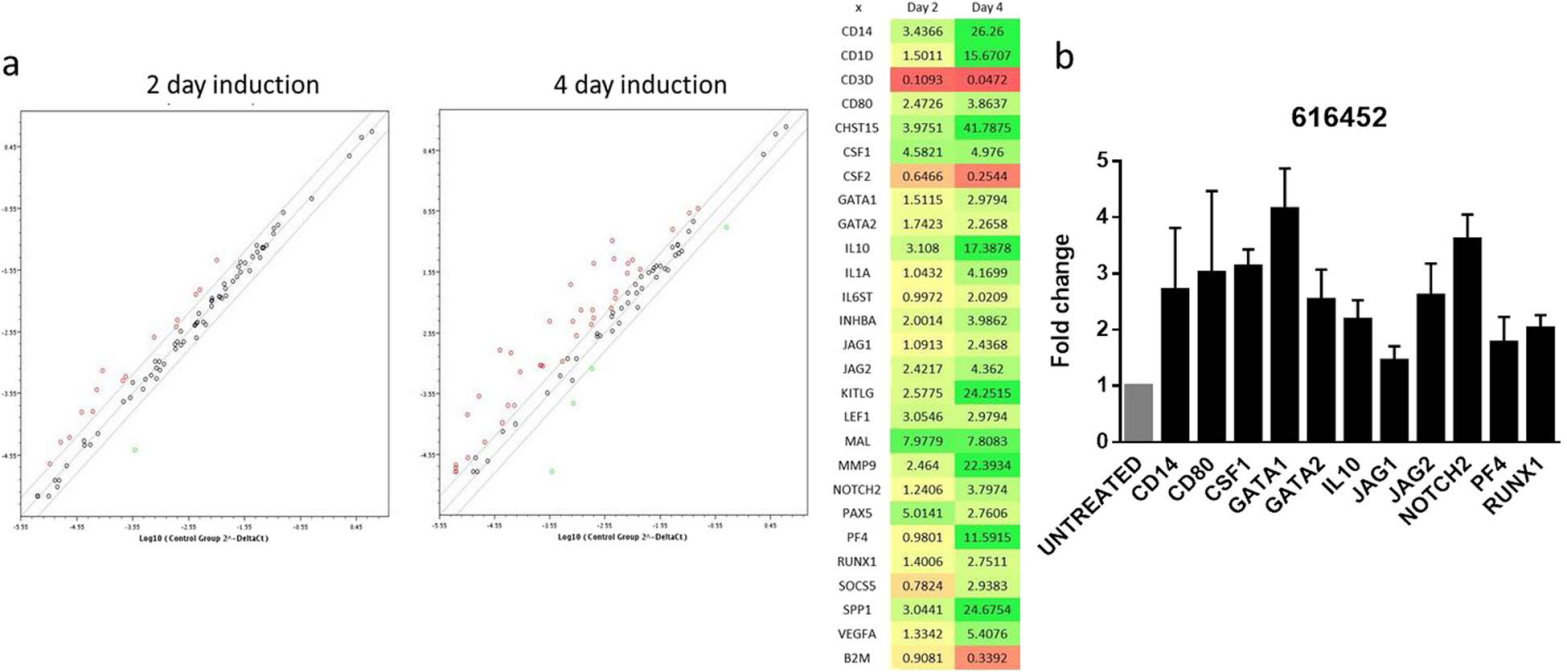
Gene expression profile and RT-PCR of cells after 4 days of induction. Gene expression profile of CB CD34^+^ cells induced for 2 and 4 days compared to control (**a**). There are noticeably more genes expressed and at higher levels on 4 days of induction compared to that of day 2. There are 18 genes total upregulated on day 2, and by day 4, there were 38. On the right is a heat map illustrating the values of expression in terms of fold increase over control. *Green* is indicative of upregulation while *red* is downregulation. Quantification of the expression level of related genes in human umbilical cord blood cells treated with or without 616452 harvested at day 4 by quantitative PCR (**b**). 616452 untreated umbilical cord blood cells were used as controls as shown in the figure. The values were normalized by GAPDH expressed as relative quantitation (RQ), the *error bars* depict the SD (*n* = 3)

Real-time PCR (RT-PCR) examined the expression of a focused panel of 81 genes (Table 1) related to hematopoietic cells (Qiagen’s PAHS-054Z hematopoiesis array) in vehicle and chemical-treated CD34^+^ cells from human cord blood. Since maturation does not visually occur until day 4 of induction, cells induced for 2 and 4 days were compared to un-induced controls (Fig. 6a). There was a significant difference between 2 and 4 day-induced cells with about 15 genes upregulated and 1 downregulated on day 2, and 38 genes upregulated and 4 genes downregulated on day 4. Most noticeably, the genes associated with megakaryopoiesis that is related to TPO-independent pathways (Notch1 or 2, GATA1, GATA2, iL1, iL6, iL10, and SCF) were up regulated. Repeat RT-PCR study further confirmed the significant fold increases in CD14, CD80, CSF1, GATA1, GATA2, IL10, JAG1, JAG2, NOTCH2, and PF4 which may give clues to the mechanism of action mainly related with TPO-independent pathways (Fig. 6b).

**Table 1:** Genes studied under the effect of 616452

ANGPT1	CD2	CD8A	FLT3LG	IL10	INHA	LRMP	PAX5	STAT1	VAV1
APC	CD27	CEBPE	FUT10	IL11	INHBA	MAL	PECAM1	STAT3	VEGFA
ASH2L	CD34	CEBPG	FZD1	IL12B	JAG1	MAP4K1	PF4	STIM2	WNT3A
BLNK	CD3D	CHST15	GATA1	IL1A	JAG2	MMP9	PTPRC	TAL1	ACTB
CBFB	CD3G	CSF1	GATA2	IL2	KDR	NCOA6	RBPJ	TEK	B2M
CCR1	CD4	CSF2	HDAC4	IL20	KIT	NOS2	RUNX1	TLR3	GAPDH
CD14	CD44	DLL1	HDAC5	IL25	KITLG	NOTCH1	SFXN1	TLR4	HPRT1
CD164	CD80	ETS1	HDAC7	IL31RA	LEF1	NOTCH2	SOCS5	TNFSF11	RPLP0
CD1D	CD86	ETV6	HDAC9	IL6ST	LMO2	NOTCH4	SPP1	TRIM10	HGDC

Table of the 81 genes analyzed under 2 and 4 days of induction

#### 616452 induces human platelet production in NSG mice

**Fig. 7:**
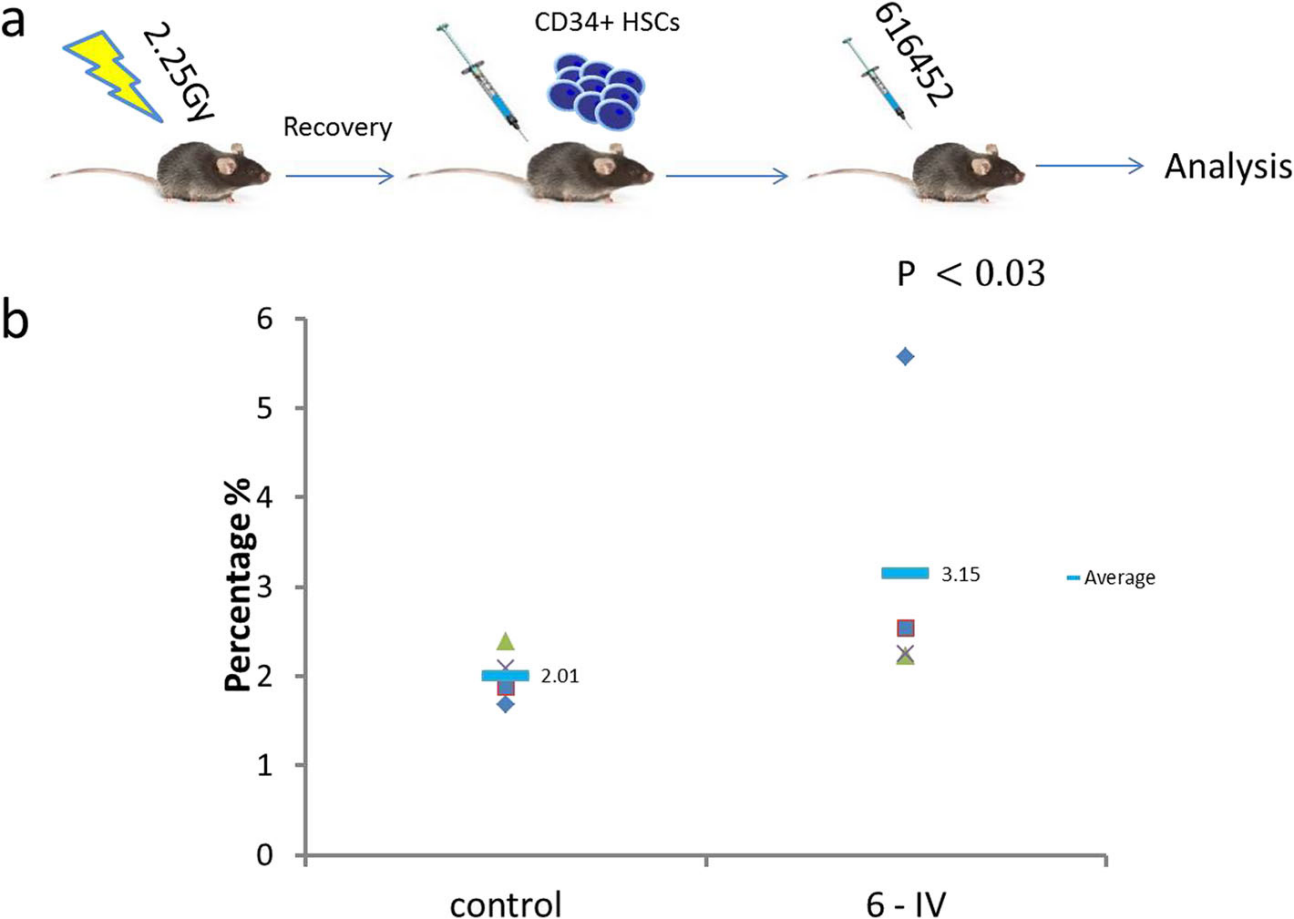
616452 effect in mice. Twelve 10-week old mice were sublethally irradiated and transplanted with human CB CD34^+^ and then given either control injections or iMK injections at 15mg/kg (**a**). Platelet analysis was done on day 8 (**b**) and was found to have significantly increased platelet counts compared to control

Several animal models were tested, but the main model used looked at the ability of CD34^+^ cells to differentiate mature and secrete platelets (Fig. 7a). Twelve 10-week-old NSG mice were given irradiation and subsequently followed by CD34^+^ transplantation. The cells were allowed to home into their niche for 3 days prior to the daily injections of 616452 chemical at 15 mg/kg via intraperitoneal (IP) or intravenous (IV) injections. Injections lasted for 12 days. On day 8, the mice were analyzed for human platelets using a tail bleed and were found to have significant increases of at least 50% in human platelets compared to that of control, induced with DMSO/PBS solution (Fig. 7b). Analysis on day 12 of the mice’s peripheral blood and bone marrow revealed a slight increase in MK engraftment via hCD41^+^ in the BM and little to no difference in the platelet measurements. IP injections were found to have little to no effect compared to IV likely due to first pass metabolism of the drug.

#### Statistical analysis

Results are presented as the mean +/−the standard deviation. Statistical significance was confirmed using unpaired student’s *t* test, Mann-Whitney test, or analysis of variance.

## Discussion

There is a large difference between cells isolated from BM and those from CB. In vitro culture of BM CD34^+^ cells results in small percentage (<1%) of cells becoming large megakaryocytes by morphology. However for cord blood, there is a significant delay with no large cells appearing at all in the first 12 days of culture. This delay is of significant importance as it introduces many platelet-derived issues during CB transplantations, which are frequently associated with a late recovery and engraftment of peripheral blood cells of donor origin as compared to transplantation of BM or mobilized PB. As a result, patients present with thrombocytopenia and delayed hospital stays due to insufficient platelet counts. To date, the major proponent used in the development and maturation of megakaryocytes is thrombopoietin (TPO) which was used in all our experiments as the control.

We initially discovered 616452 when CD34^+^ cells induced with it produced a significant number of large megakaryocyte-like cells. Within four days of induction, there was an abundance of megakaryocytes, and they only continued to increase in size. These large cells were 2–16 times the size of the typical cells in the control. In fact, the control cells when induced with only TPO had little to no observable increase in cell sizes. Cell cultures typically lasted only eight days after induction due to aggregation and formation of MK-burst forming colonies (Fig. 4i–l). Analysis of cells after day 8 resulted in inconsistent results due to the nature of colonies as well as ease in which the MKs could be lysed by simple pipetting techniques.

These large cells were identified by their morphology, phenotype, and functionality. A Giemsa-Wright stain displayed typical megakaryocyte morphology of lobular and multi-nucleated nucleus with a granular cytoplasm. Analysis by flow and live cell staining revealed these large cells to be CD41 positive, a protein typically expressed on platelets and megakaryocytes. Additionally, the induced cells were found to have drastically increased nuclear DNA content compared to control with cells developing ploidy numbers as great as 64 N within 8 days of induction.

Between experiments (>5), there was an inconsistency in the number of large cells among the samples from different patients. While the CD41 population appeared to increase regardless of the patient, the population seemed to differ drastically between certain patients. This was likely caused by the variation of megakaryocyte precursors among patients. Using flow cytometry, we sorted the cells for CD41^+^ and CD41^−^ cells. Both populations were then induced using the small chemical and TPO. We found that the CD41^+^ population induced with the chemical had increased ploidy of 4 N or greater in over 50% of the cells while the control cells only had approximately 6%, an increase of nearly 10-fold. In the CD41^−^ population, there was only an increase in 3% of cells of 4 N or greater ploidy compared to non-induced. While not perfect, a small fraction of CD41^+^ cells or hematopoietic stem cell remained after sorting that developed into the MK lineage explains the slight increase in the number of ploidy cells.

Often associated with megakaryopoiesis, one concern was the dependence of this chemical on the presence of or priming by, in the case of CD41^+^ cells, TPO. CB CD34^+^ cells were induced with the chemical for 8 days in the presence and absence of TPO, and it was found that the increase of MK maturation increased in nearly all conditions, with or without TPO (Additional file 2: Figure S2g). However, the presence of cytokines does increase overall cell counts and copy number of MK genes, which produces larger numbers of MKs.

The hematopoietic stem cells (HSCs) of various other species such as monkey and mouse were isolated and induced with the same chemical. A comparison against human HSCs revealed the chemical to be the most potent in monkey with a 30–200% increase in ploidy development of certain sizes and nearly 2000%-fold greater than that of mouse HSCs (Additional file 3: Figure S3). Even so, each species still experienced an increase in ploidy development.

The efficacy of this small molecule on platelet production was initially tested in various mouse models to no avail. It was found that the delivery of this chemical was of great importance. When given via intraperitoneal (IP) injections, the mice experienced no effect and in fact had a negative impact on the mice. The optimal mode of delivery was via intravenous (IV) injections. Many mice models failed initially, but after the discovery that mice HSCs are significantly less impacted by the chemical than that of human or monkey HSCs, a new transplant model was developed. The mice were given a sublethal dose of irradiation at 2.25 Gy to make room for engraftment and then injected with 50000 CD34^+^ cells and 300000 progenitor cells (CD34^+^ cells cultured for 4 days). The mice were then given 3 days of rest to allow the cells to hone into their respective niches after which each mouse was administered one daily dose of the chemical at 15 mg/kg. On day 8, we found that human platelet production in the induced mice were 50% greater than the control mice.

The concentration of 616452 used varied from 2.5–40 uM. Although there appeared to be potent effects at higher concentrations, they could be difficult to achieve in vivo. The side effect profile could be of great concern as mice injected with 50 mg/kg showed significant toxicity (data not shown) with the majority of the mice dying before the conclusion of the in vivo assay. This side effect profile is of great concern when thinking about the translational effect to clinical medicine, and therefore further studies on the toxicities and delivery mechanisms by which to reduce such toxicities are necessary.

## Conclusion﻿﻿

616452 is a TGF-β inhibitor that provides a novel pathway for the induction of megakaryocytes that do not work like the SRC kinase inhibitors like diMF or SU6656 or any Aurora A Kinase inhibitors. Unlike other compounds, 616452 does not have much in vitro toxicity at the concentrations tested in this manuscript and is able to enhance the differentiation into megakaryocytes and speed their maturation. This small chemical provides a new approach toward megakaryocytic diseases that has the potential for clinical treatments without unwanted side effects of other inhibitors caused by their toxicity to all cells.

## Additional files

Additional file 1: Figure S1. 616452 induces megakaryopoiesis in human cord blood (CB) CD34^+^ cells. Chemical induction of CB CD34^+^ using 616452 (10 uM) for 8 days compared to control (**a**). Cells were analyzed by flow cytometry for CD41 on day 5 (**b**) and also stained using a Giemsa-Wright Stain after 8 days culture to illustrate the granular cytoplasm, and the multi-nucleated and lobular nature of the nuclei (**c**). (JPG 146 kb)
Additional file 2: Figure S2. 616452 and TPO independence and synergy. BM and CB CD34^+^ cells were cultured in the presence of SCF and TPO. In 4 days of culture, the cells were all healthy and relatively the same size (**a**,**b**). By 8 days of culture, BM cells appeared to have a few (<1%) megakaryocytes with a size approximately four times that of a regular HSC **(c).** CB however, had no such cells (**d**). Those cells induced with 616452 for both BM and CB developed a significant number of large MKs (**e**,**f**). When CB CD34^+^ cells were induced with 616452 for 8 days under various cytokine conditions (**g**), it was found that 616452 works independently of either SCF or TPO inducing MK maturation under all conditions. (JPG 193 kb)
Additional file 3: Figure S3. 616452 on monkey BM CD34^+^ cells. Monkey CD34^+^ cells were purchased from Lonza and mouse HSCs were isolated using ckit + MACs isolation kit. Human CD34^+^ were isolated from cord blood. Cells were cultured for 6 days with 616452 or DMSO for 6 days prior to analysis via propidium iodide staining. (JPG 37 kb)
