# Supplementary figures and images for: Identification of a potent small molecule capable of regulating polyploidization, megakaryocyte maturation, and platelet production
Source: J Hematol Oncol. 2016 Dec 8;9:136. doi: 10.1186/s13045-016-0358-y (PMC5143458; doi:10.1186/s13045-016-0358-y)

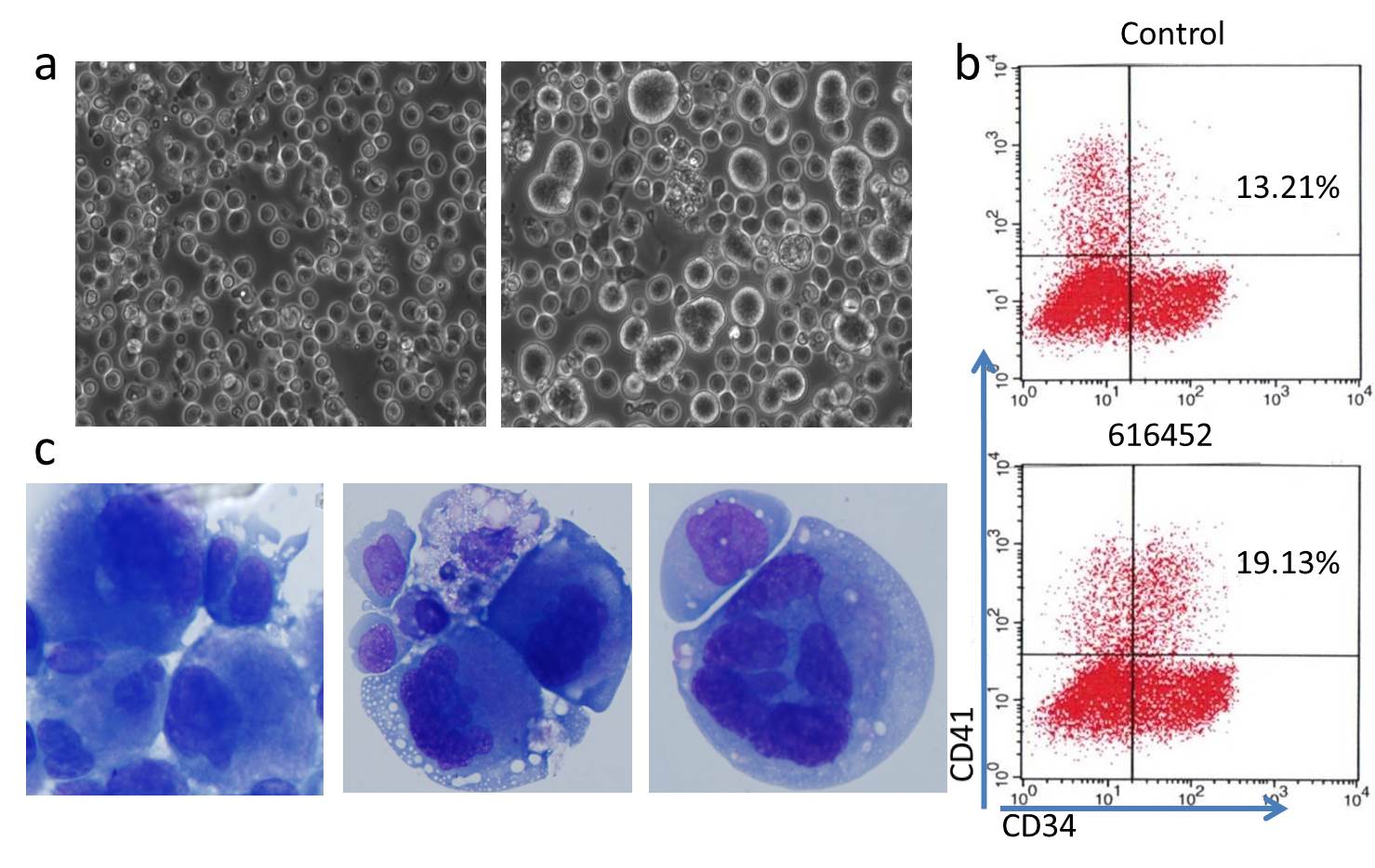

Supplement: Additional file 1: Figure S1. — 616452 induces megakaryopoiesis in human cord blood (CB) CD34+ cells. Chemical induction of CB CD34+ using 616452 (10 uM) for 8 days compared to control (a). Cells were analyzed by flow cytometry for CD41 on day 5 (b) and also stained using a Giemsa-Wright Stain after 8 days culture to illustrate the granular cytoplasm, and the multi-nucleated and lobular nature of the nuclei (c). (JPG 146 kb) [file 13045_2016_358_MOESM1_ESM.jpg]

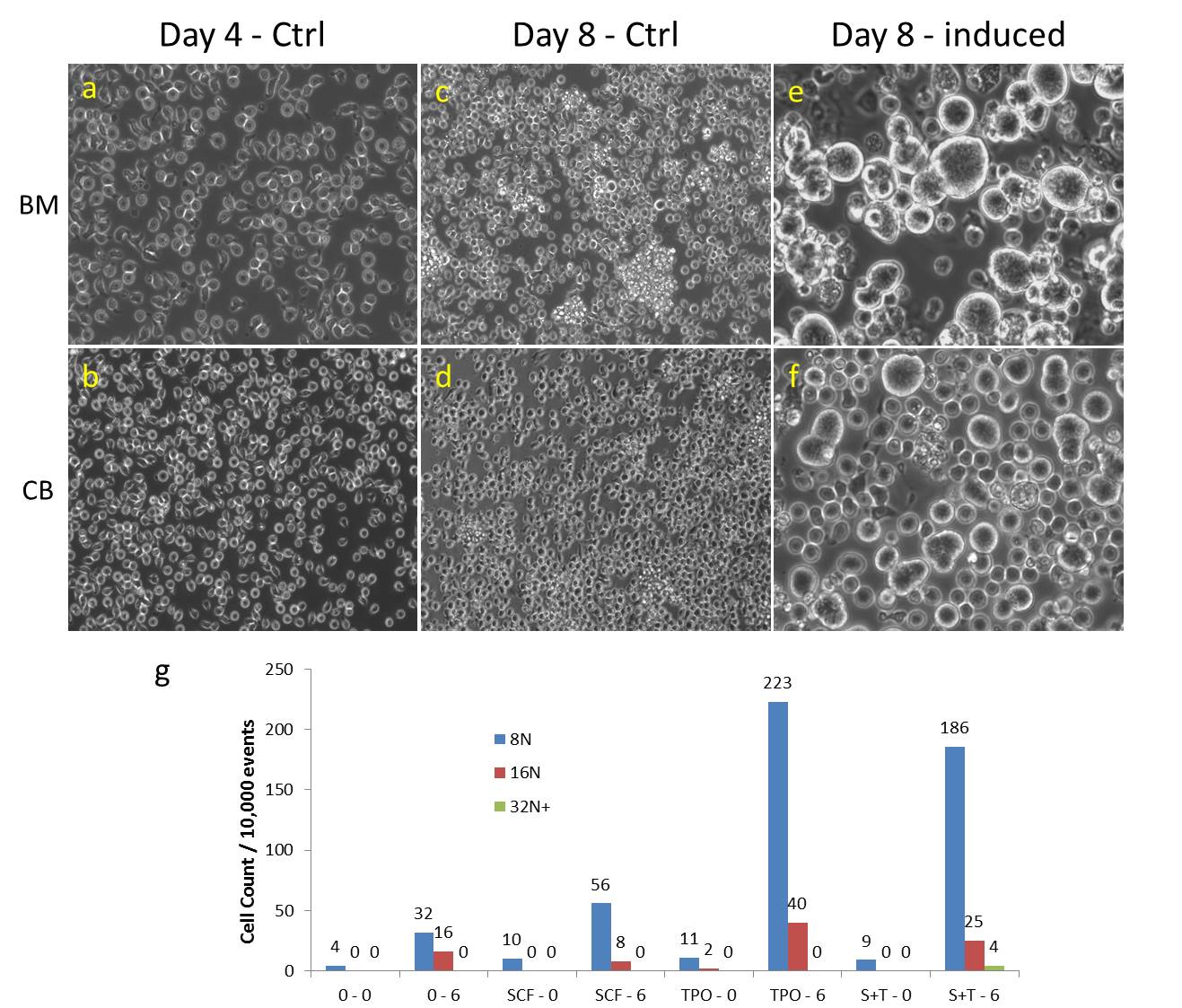

Supplement: Additional file 2: Figure S2. — 616452 and TPO independence and synergy. BM and CB CD34+ cells were cultured in the presence of SCF and TPO. In 4 days of culture, the cells were all healthy and relatively the same size (a,b). By 8 days of culture, BM cells appeared to have a few (<1%) megakaryocytes with a size approximately four times that of a regular HSC (c). CB however, had no such cells (d). Those cells induced with 616452 for both BM and CB developed a significant number of large MKs (e,f). When CB CD34+ cells were induced with 616452 for 8 days under various cytokine conditions (g), it was found that 616452 works independently of either SCF or TPO inducing MK maturation under all conditions. (JPG 193 kb) [file 13045_2016_358_MOESM2_ESM.jpg]

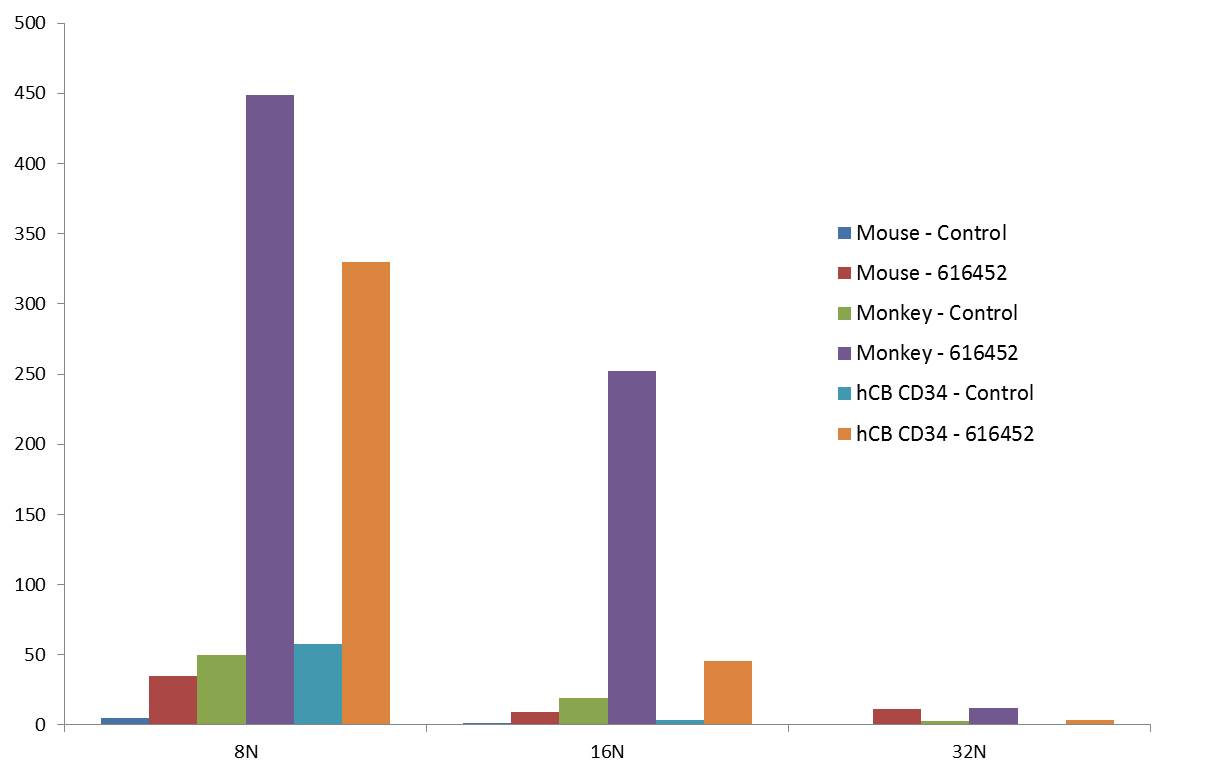

Supplement: Additional file 3: Figure S3. — 616452 on monkey BM CD34+ cells. Monkey CD34+ cells were purchased from Lonza and mouse HSCs were isolated using ckit + MACs isolation kit. Human CD34+ were isolated from cord blood. Cells were cultured for 6 days with 616452 or DMSO for 6 days prior to analysis via propidium iodide staining. (JPG 37 kb) [file 13045_2016_358_MOESM3_ESM.jpg]
